# A challenging entanglement: health care providers’ perspectives on caring for ill and injured tourists on Cozumel Island, Mexico

**DOI:** 10.1080/17482631.2018.1479583

**Published:** 2018-06-05

**Authors:** Leon Hoffman, Valorie A. Crooks, Jeremy Snyder

**Affiliations:** a Department of Geography, Simon Fraser University, Burnaby, Canada; b Faculty of Health Sciences, Simon Fraser University, Burnaby, Canada

**Keywords:** Cozumel Island, Mexico, tourism, care provision, challenges

## Abstract

**Purpose**: Despite established knowledge that tourists often fall ill or are injured abroad, little is known about their treatment. The intent of this study was to explore health care professionals’ treatment provision experiences on Cozumel Island, Mexico. **Methods**: 13 semi-structured interviews were undertaken with professionals across a number of health care vocations on Cozumel Island. Interviews were transcribed and thematically analysed to determine common challenges faced in the provision of treatment for transnational tourists. **Results**: Three thematic challenges emerged from the data: human and physical resource deficiencies, medical (mis)perceptions held by patients and complexities surrounding remuneration of care. Health care providers employ unique strategies to mitigate these challenges. **Conclusion**: Although many of these challenges exist within other touristic and peripheral spaces, we suggest that the challenges experienced by Cozumel Island’s health care professionals, and their mitigation strategies, exist as part of a complex entanglement between the island’s health care sector and its dominant tourism landscape. We call on tangential tourism services to take a larger role in ensuring the ease of access to, and provision of quality health care services for tourists on Cozumel Island.

## Introduction

In the classic sense, tourism, as time and space apart from the everyday (Franklin, 2003), offers promises of rejuvenation and rehabilitation of mind and body (Richards, 1996). However, tourism is also replete with perceived and real risk (Williams & Baláž, 2014; Yang & Nair, 2014) that can lead to unfavourable health outcomes for those travelling abroad. Such consequences of travel are finely detailed within a wealth of travel medicine scholarship. Unfortunately, this body of literature offers sparse consideration of the health care services provided in destinations. This is especially true of tourists’ engagement with health care professionals, for whom the everyday can involve caring for ill and injured travellers. This article begins to fill this gap by examining the perspectives of health care workers on Mexico’s tourism-dependent Cozumel Island, as they provide treatment for tourists in need of urgent medical attention. Specifically, we examine challenges faced by providers in delivering care, including resource deficiency, (mis)perception of the island’s health care sector, and complexity regarding service remuneration. Finally, we contextualize these challenges as part of a complex interplay between the health care sector and touristic aspirations of Cozumel Island.

Pathologies of injury and illness on holiday are comprehensively documented. Typically, studies track specific spatio-temporal disease, illness and injury vectors that commonly affect tourist populations (e.g., Chen, Chang, & Chen, 2014; Leshem et al., 2016; Ratnam, Leder, Black, & Torresi, 2013), and discuss associated pathological concerns (e.g., Flores, Hickey, Fields, & Ottolini, 2015; Matteelli, Centis, Sulis, & Tadolini, 2016; Salazar-Austin et al., 2015). Comprehensive statistical data about tourists’ use of health care abroad and their health outcomes is, however, mostly sparse and dated (Angelo, Kozarsky, Ryan, Chen, & Sotir, 2017). That which does exist suggests that tourists are at an elevated risk of injury, illness or death compared to both those who remain at home (Steffen, deBernardis, & Baños, 2003) and local populations in tourist destinations (Bauer, Körmer, & Sector, 2005; Mitchell, Williamson, & Chung, 2011). Two reviews respectively state that 75% (Steffen et al., 2003) and 65% (Hill, 2006) of travellers to developing countries report a degree of health impairment while abroad. More recently, a study by Vilkman, Pakkanen, Lääveri, Siikamäki, and Kantele (2016) revealed that health care was sought by 10% of overseas tourists and as many as 79% would go on to report that they fell ill while travelling. Further, in contrast with local populations, tourists are believed to be at higher risk of injury due to motor vehicle collision (McInnes, Williamson, & Morrison, 2006), and across all types of unintentional injury, are likely to experience more severe injuries (McInnes et al., 2006; Steffen et al., 2003). While few reviews of cruise tourist morbidity exist, a 1991 review of 172 cruises found that visits to the ship infirmary were made by 3.6% of passengers, and of those visiting the infirmary, at least 2.8% were disembarked before the completion of the cruise (Peake, Gray, Ludwig, & Hill, 1999). Although these data are dated, one can assume a substantial increase in contemporary demand for health care services considering that the global number of cruise passengers has grown over 550% between 1991 and 2016 (Cruise Market Watch, 2017).

Outside of epidemiological work, some existing literature considers the health risks of travel (e.g., Jackson & Abubakar, 2017; Lüthi & Schlagenhauf, 2015), pedagogical approaches to better inform and prepare tourists for such risks (e.g., Marchand, Merrina, Gagnayre, & Bouchaud, 2017; Seale et al., 2016), and outcomes of this education (e.g., Angelin, Evengård, & Palmgren, 2014; Croughs, Remmen, & Van den Ende, 2014). Such work aims to contextualize the assumption that a confluence of “appropriate pre‐travel advice and patient compliance are … key elements in ensuring that world travelers return home in good health” (McIntosh, 2015, p. 143). However, there is no consensus as to the efficacy of pre-travel health advice (Angelin et al., 2014; Wieten, van der Schalie, Visser, Grobusch, & van Vugt, 2014), with some research reporting high rates of injury or illness despite visits to travel medicine clinics before departure (Santantonio et al., 2014). Researchers suggest this may be the outcome of inadequate training of health care professionals (Kogelman et al., 2014; Leder, Bouchaud, & Chen, 2015) or a lack of confidence in dispensing pre-travel advice (Bascom, Rosenthal, & Houle, 2015). Conversely, tourists may struggle to retain and recall information when needed (Angelin et al., 2014; Bauer, 2006) and can be non-compliant with or actively disregard advice received (LaRocque & Jentes, 2011; Schwartz, LaRocque, & Ryan, 2012). Further, data suggests that the majority of travellers do not actively seek out pre-travel health advice (Baer et al., 2014; Kogelman et al., 2014) and are often unaware of the health risks posed by travel destinations, a factor that can leave tourists unprepared for emergent health care complications.

Within travel medicine research there is very little engagement with health care professionals in destination countries, offering thin details about their experiences of providing treatment to patients who are on holiday, including the challenges they face. Studies that do exist suggest that challenges may include lack of patient knowledge, communication difficulties, and assurances of personal safety for physicians (Fitzwater, 2008). Outside of traditional tourism research, examination of medical tourism (i.e., intentional travel abroad to privately access health services) has provided some insight into care for international patients. Here, researchers have offered important insights regarding the potential drawbacks and benefits of care abroad. These include concerns about the safety and quality of medical care outside of one’s home country (Gan & Frederick, 2015; Hudson et al., 2016), language and cultural tensions with health workers abroad (Hudson et al., 2016; Whittaker & Chee, 2015), and inadequate continuity of care due to disruptions in the continuing medical record (Martínez Álvarez, Chanda, & Smith, 2011).

Most existing research regarding tourists’ health care use while in Mexico—the country of focus in this article—examines USA (US) residents, typically of Hispanic origin (Wallace, Mendez-Luck, & Castañeda, 2009), who undertake short trips across the border for medical or pharmaceutical care (Byrd & Law, 2009; Su, Pratt, Stimpson, Wong, & Pagán, 2013; Su & Wang, 2011). Reasons for this cross-border movement include lower priced products and services, easier access to care, and cultural familiarity (Horton & Cole, 2011; Su & Wang, 2011; Wallace et al., 2009). Although there are no in-depth qualitative studies to evaluate patient care, surveys do suggest high satisfaction with care among these populations (Byrd & Law, 2009). Elsewhere, a small collection of literature engaging with lifestyle and retirement migrants provides insight into how some non-Mexicans characterize health care they have accessed while in the country. Here, Mexico’s private health care is typically characterized as lower priced, offering a diversity of quality services, as well as a sense of personalization and empathy not found in international patients’ home countries (Amin & Ingman, 2010; Hoffman, Crooks, & Snyder, 2017; Sloane, Cohen, Haac, & Zimmerman, 2013; Sunil, Rojas, & Bradley, 2007). This literature also identifies challenges for patients, including concerns about the quality of products and services, inability to access certain technologies, complications in decision-making, and insufficient health insurance coverage (Hoffman et al., 2017; Sloane et al., 2013)

Cozumel Island is Mexico’s largest eastern island and is home to approximately 85,000 people (Instituto Nacional de Estadística y Geografía (INEGI), 2015). Cozumel is expected to host 3,566,700 cruisers aboard 1,160 ships in 2017, making it the most popular cruise destination in the Western Caribbean (CruisePortInsider, 2017). Outside of cruise tourism, approximately 500,000 overnight tourists alight each year (Secretaría de Turismo del Estado de Quintana Roo (SEDETUR), 2015), establishing the island as a site of fluctuating and converging transnational mobilities. Unfortunately, the extent to which tourists access medical care on Cozumel is not known. However, the island does boast the presence of two public and four private hospitals, amongst numerous ancillary health care services such as dental offices, hyperbaric clinics (Enjoy Corporation, 2017; TripAdvisor, 2017), and pharmacies (Hoffman, 2017), the numbers of which suggest that there exists no shortage of injured and ill vacationers in need of medical attention. With the treatment needs of vacationers in mind, here we present the findings of semi-structured interviews with health care workers on Cozumel who routinely treat these travellers at local hospitals, medical centres, and dental clinics. Drawing on the findings of our thematic analysis of interviews with local health care providers, we specifically identify the challenges they face in treating this short-stay, relatively mobile patient group. In doing so we contribute to the growing area of research situated at the nexus of tourism and health while also providing insights that can be used to identify meaningful interventions to assist with facilitating treatment of this patient group.

## Methods

This analysis contributes towards a broader, exploratory case study of transnational health and health care mobilities on Cozumel Island, Mexico, which was specifically chosen as a site of converging, internationally mobile populations and conglomerate health care services. While our broader case study includes significant observational fieldwork and engagement with Cozumel Island’s international lifestyle and retirement migrant population, this analysis focuses upon semi-structured interviews with health care professionals who provide medical treatment for ill and injured tourists on the island.

### Data collection

Prior to data collection, we received ethics approval from the Simon Fraser University Office of Research Ethics. Two research visits to Cozumel Island were conducted by the first author: one from March-April 2015 (accompanied by the second author), and the other from January-February 2016, where face-to-face interviews were carried out with health care providers. While this study is inherently exploratory and thus it is not appropriate to limit participation to a set group, we employed purposive sampling to prioritize participation by senior administrators with clinical oversight and clinical staff directly involved in the treatment of tourists. This allowed us to tailor recruitment towards participants with decision making capabilities, English language competency, and appropriate levels of experience and knowledge (Etikan, Musa, & Alkassim, 2016). In the majority of cases, small facility size afforded little division between administrative and clinical positions, with many providers filling dual roles. While our goal was to recruit at last one participant from each of the island’s internationally focused facilities, we employed a temporal sampling strategy only limited by the period of data collection. With confined time in the field, recruitment thus remained continuous throughout each visit to Cozumel Island. Participants were recruited via a standard recruitment email (with addresses being obtained from facility or clinic websites) or by approaching health care establishments on foot. In most cases, both email and in-person recruitment strategies were undertaken in order to alleviate potential language complications, ensure participant appropriateness and provide effective communication of our study goals and details regarding participation. While snowball sampling was not specifically employed, the close-knit nature of the island’s health care sector often led participants to suggest colleagues they believed might be able to offer valuable insight. Those who expressed interest in participating were provided with a detailed study information document as well as information concerning participant rights and our ethics approval. Following initial contact, subsequent telephone or email communication enabled participants to select a time and place of their choice to complete the interview.

Data collection was conducted via semi-structured face-to-face interviews as they allow for a pragmatic approach towards the exploration of little known topics, offering flexible examination of participant subjectivities and the ability to query emergent themes (Corbin & Morse, 2003). Interviews ranged from 35 minutes to 2 hours in length, and followed an interview guide created in recursive collaboration between this study’s investigators. Questions probed participants’ experiences providing treatment for tourists and other international patients, including caring practices (e.g., can you tell me about the care you offer for international patients? What is the typical experience involving an international patient?), administrative procedures (e.g., does the treatment of international patients offer different administrative challenges?), interacting with patients (e.g., do you think that international patients have different expectations of health care services than local patients?), and expectations for the future of health care on Cozumel (e.g., what do you see for the future of this clinic and for health care on Cozumel as an island and tourist destination?).

### Data analysis

All interviews were conducted in English and transcribed verbatim by the lead author. Following transcription, each author independently reviewed six transcripts. After several rounds of discussion and collaborative exploration, we identified the challenges of treating vacationers to be an important emerging issue that warranted investigation via thematic analysis. Following this, the lead author undertook multiple reads of the transcripts to identify a number of pertinent meta-themes, and, using QSR NVivo, coded thematically with input from the other investigators regarding the coding scheme and its interpretation. Coded extracts were then shared amongst the authors to confirm the emergent challenges and finalizing the scope of each meta-theme, data interpretation, and determining inclusion in the final analysis. A collaborative approach to discussion, exploration and analysis of the data, as well as preparation of the interview guide, allows for investigator triangulation. This, alongside multiple site visits, detailed field notes as an audit trail and awareness of researcher positionality and subjectivity help to enhance the rigour (Bradbury‐Jones, 2007; Wolfram Cox & Hassard, 2010), validity and reliability of the study (Long & Johnson, 2000).

## Results

In total, 15 health care workers participated in 13 interviews (two were conducted in pairs). Those interviewed had collectively interacted with hundreds of tourist-patients and had thousands of hours of clinical and senior administrative experience between them. Participants, working across a range of health care professions, represented a number of establishments including private hospitals or clinics, dental offices, and medical authorities at port agencies. As noted above, participants’ duties typically fell across both administrative and clinical responsibilities. The duration of participants’ employment within Cozumel’s health care sector ranged from 2.5 months to over 32 years, with the majority having worked elsewhere in Mexico for numerous years prior to working on Cozumel.

Trauma and age-related morbidities were stated to be the principal reasons that tourists require medical attention on Cozumel. Motor vehicle collisions were said to account for most traumatic injuries, with risk heightened by inexperience with local roads and traffic, as well as indiscriminate alcohol consumption. As one participant explained, “people come from the cruise ships, they rent a moped or a motorcycle and they go around the island and they drink, so there’s a lot of accidents, a lot!” Age-related morbidities were commonly attributed to older cruise ship passengers, and included trauma as well as managing exacerbations of existing chronic illness, outcomes of medication non-adherence, and most commonly, cardiac events. It was not uncommon for passengers to be admitted to health care facilities directly from cruise ships.

Although Cozumel’s private hospitals and clinics specialize in addressing the health care needs of the large number of tourists who visit the island each year, the interviews revealed that this does not preclude numerous challenges in treating this patient group. In the remainder of this section we examine three key challenges identified by participants that emerged from thematic analysis: resource deficiencies, medical (mis)perceptions, and remuneration complexities. We also identify solutions or strategies to mitigate these challenges raised by the participants.

### Resource deficiencies

Although the island does not suffer from a dearth of health care infrastructure, participants revealed that Cozumel’s hospitals often lack specialist human resources or specific equipment to provide essential treatment for ill and injured visitors. As one participant stated, “you have limitations [on Cozumel] because you only have basic specialties, there is no high speciality here.” This is exacerbated by difficulties in attracting specialists to the island: “[one of] our challenges … is bringing more doctors to the island, more specialities” such as oncologists, who are flown in as needed. Recruitment and retention of nursing staff was considered similarly frustrating: “[being an] island, [it] is very difficult to bring, for example, nurses, especially nurses … we need to pay more because they don’t want to be, to be [on] an island. They say … [after] only almost three months [that] it’s boring to live here,” revealing the extent to which the realities of managing everyday life on a small island can influence Cozumel’s health human resources pool.

There was also concern regarding deficiencies in needed material health resources, including medical technologies and consumables such as blood products, on the island. Participants agreed that while some hospitals are better equipped than others, noted in statements such as “CostaMed is the facility that is better equipped … in Cozumel,” the island’s health care facilities typically lacked the medical technologies needed to perform more complicated diagnostic procedures and treatments. In comparison to Mexico’s mainland, one participant noted that when considering “the equipment for … very big surgery, some brain tumours or open surgery of the chest for the heart, very high procedures, it’s a fact that in Cozumel we don’t have that sort of equipment.” For many, both financial constraints and available space were prohibiting factors for obtaining desired medical equipment. Further, participants commonly lamented the lack of a comprehensive biofluid facility with storage for blood and its component parts, as well as other essential bodily fluids. As a participant explained, while “[the state hospital] have blood and they have plasma, that’s all, you don’t have platelets, you don’t have … any kind of blood compliments [sic] that help … a patient survive.” Generally, participants felt that with the closest facility being located on the mainland, time delays in obtaining necessary biofluids could elevate the risk of negative health outcomes for vacationers in need of medical attention.

While the above deficiencies in human and material resources within Cozumel’s health care landscape reveal challenges for treating ill and injured vacationers, participants explained that these challenges can be met through collaborative efforts or circumvented entirely through patient evacuation. One participant stated that it is a legal requirement for each hospital to “have [a] liaison with some of the [other] hospitals. This should be a contract, each hospital should have a contract with two other hospitals.” For example, this participant noted that “[at] this moment I don’t have a [specific diagnostic machine] so I bring the patient to [another clinic]” where that machine is available, revealing a collaborative effort in ensuring essential access to diagnostic resources. Resource pooling between hospitals was considered essential. One participant noted that informal collaborative agreements exist “between doctors … if a patient is ill we call each other, even … if he’s working at another company, … [and] sometimes we lend these hospitals, even the state hospital, equipment.” Such resource sharing does not happen without concern. For example, a participant explained that there can be “differentiation [in] … the safety measures in this hospital … My practice needs to be very safe, and I know the other safety measures in other hospitals [and they are] … not enough.” Another way of coping with resource deficiencies is by evacuating patients to mainland Mexico or even the US. However, participants understood that even “a very well-equipped air ambulance [can present] difficult moments for the medical team, and obviously for the patient” who may remain in a vulnerable state. For this reason, evacuations are typically considered to be a last resort unless they are specific demands of patients’ insurers or at the express wishes of the patient or their family.

### Medical (mis)perceptions

Challenges can also arise due to patients’ preconceived perceptions about the state of health care in Mexico, often aligning it with wider assumptions of danger, crime, and poor practice. Ill and injured vacationers can be reluctant to receive care, leaving health care professionals the task of legitimating Cozumel’s health care facilities and services. Noted by one participant, tourists “don’t trust in the … medical expertise of our doctors, because it’s Mexico, they have that … thinking that Mexican medicine is not what [acceptable care] should be. But it is, it’s really good.” This belief may be perpetuated by stereotypes shared by the media or among friends:

When [the patients] are going home, they say “I am so sorry, I didn’t know, you know? You always hear these horrible stories about Mexico, that a friend of a friend went to Tijuana and got their eyes out or they took their kidney.” And that’s what they think, that all the doctors in Mexico are the same and that’s all they do, you know?

Patients may also consider Mexican health care competencies to be underdeveloped. One participant stated that “when you’re in [Mexico], you’re afraid to get services there because of what people say [about the country] … You have a broken bone, you’re gonna say ‘oh, in Cozumel, they gonna kill me, they are like third world medical attention’.” Similarly, patients may also “think that because they are out of the USA … [in a] country of Latin America, … [practitioners] do not have the resources to attend [patients] properly, or the water [or] food is contaminated and they reject … the medical treatment.” Further, health care does not escape broader issues regarding race; one participant noted that there are “even issues of nationalities: ‘I don’t want to be treated by Mexicans, I don’t want to have [a] blood transfusion [from] Mexican people’,” revealing the extent to which cultural prejudices and perceptions can create challenges for treatment.

Cultural biases and stereotypes also precipitate vacationers’ expectations about the aesthetics of quality health care. Knowing this, one participant explained that it is important to “[show that] we can do the procedures, [that] we have all the equipment and materials … the patients or the parents of the patients want to see it to prove that we have everything.” Another participant noted that patients want to see specific visual details, comparable to health care spaces at home, that legitimize health care in Cozumel and reinforce its quality nature:

They’re very worried about the facility. How do you see the bed, the blanket, the nurse, when they see the doctor, do you look like a doctor? … You have to look like a doctor, your appearance is very important for the patient … they are used to see[ing] the doctors with a gown, with a coat. They want see that it’s a hospital, not a medical office.

The quotes above each demonstrate the extent to which care provision for tourists can be affected by practitioners’ abilities to validate medical knowledge, practices and equipment within Cozumel’s health care facilities.

Realizing that culture shock and specific preconceptions may influence the ability to provide treatment for tourists, hospitals seek to mitigate this by offering familiar contact points. At least one hospital employs “people who are American who live here [on Cozumel] … [people] who work in the clinic [and] are the first contact [for] the patients.” Hospitals thus utilize employee cultural identity to offer reassurances of safety and quell fears. Communication may also be established with health care professionals in patients’ home countries to assist in making care decisions and to provide assurances about standards of care on Cozumel. One participant stated that they will ask vacationers: “you want to talk to your doctor back in the US, or back in Canada? Give us the phone number and we’ll tell your doctor what’s your treatment, what our plan [is] with you.” Trusted medical professionals in the patient’s country of origin are seen to help with demonstrating that medical care on Cozumel is professional and safe. For participants, engagement with the familiar is part of a broad commitment to “making [patients] feel safe, making them feel comfortable and making them feel that they’ll receive the same treatment here as … any other first world country.”

### Remuneration complexities

Another challenge when treating tourists is in obtaining payment for medical services, an issue of significance considering the for-profit nature of Cozumel’s private health care facilities. Though participants stressed the importance of providing empathetic care, the island’s facilities will typically halt treatment and relinquish responsibility if assurance of payment cannot be made: “[we will] assure the patient is stable, that his life is not at risk, and stop the medical attention.” Typically, the “last thing [tourists] keep in mind when they start a trip on a cruise ship is to finish their vacation in a hospital,” and thus some may not purchase travel health insurance or have a plan to deal with any medical expenses that arise in the course of their holiday. One participant stated, “we have patients that don’t have insurance, they don’t [have] money, they don’t have anything! I don’t know what they’re doing on the ship but they don’t have anything!” Another explained that, “patients from cruise ships [often ask] ‘is the cruise ship paying for it?’ That’s something that they always ask, ‘cause I pay for my ticket and they told me that everything’s included’.” This sentiment was broadly shared among participants, with others noting “when it becomes an issue is when they don’t have insurance and they don’t bring money”, and “sometimes even there are people that have nothing to pay, they have no money for paying … and don’t have international [insurance] coverage.” In such cases administrators will require clinicians to halt all treatment, which significantly challenges all those involved in providing and receiving care.

Collecting remuneration for medical services may be significantly complicated by insurance companies. One participant stated that “it’s when the insurance company gets involved, that’s the factor that adds turmoil into the whole case.” Difficulties arise as facilities struggle to deal with the broadly disparate nature of insurance companies’ practices and policies: “every case is different because, [for example] you can have Blue Cross insurance, but in every state every policy is different.” Even internal variation within companies may create difficulties for Cozumel’s health care facilities to establish one-size-fits-all procedures for obtaining insurance payments. However, as one participant explained, remuneration difficulties do not always arise from complex factors around billing codes and treatment costs and can be as simple as working with the operational hours of the insurance company: “some [insurance] companies … don’t have personnel for emergencies during the weekend, so you have to wait until Monday to receive the guarantee of payment, … [that’s] a big risk.” Thus, remuneration difficulties are not only a matter of accounting, but can potentially introduce unnecessary risks for patients as care beyond stabilization is typically not performed until guarantee of payment is received.

Participants noted that often there is little they can do to meet challenges posed by seeking remuneration. While delays in communication with insurance companies are typically a matter of time, absence of insurance coverage or the ability to pay out-of-pocket have few remedies. In these scenarios, two processes are typically possible: asking a cruise line for assistance (which only applies to cruise passengers), or reaching out to the patient’s home country consulate office in Mexico. While highly infrequent, participants do note that cruise lines may occasionally provide coverage for patients. Although noted above that medical care is not included a cruise passenger’s ticket, one participant did state that in some cases:

[I] contact the cruise company and tell them “well, I have done what I have to do, which is stabilize this patient, and now you have to tell me what you wanna do with [them]. Do you want me to keep him here and you’re gonna come and pick him up? Do you want me to give him the rest of the treatment, or what’s gonna happen?” Most of the times the companies are really, really, good with tourist[s] and they take care of that.

However, many participants clearly explained that a patient who has been disembarked from a cruise ship for medical care is typically no longer the responsibility of the ship and this type of situation is rare. A more probable course of action is to contact the patient’s consulate in Mexico: “You have to stay in the hospital, but you don’t have money, you don’t have anyone to call, you don’t have any insurance, what do we do? [We] have to contact … [the patient’s] international consulate. [Then] someone from the consulate [will] come and say, ‘you know what, we’re going to do this’,” although no participants expanded upon the remunerative strategies undertaken by consular officials.

## Discussion

This analysis has explored the challenges associated with providing care for ill and injured tourists visiting Cozumel Island, Mexico, as understood by those working in the island’s private health care sector. While existing studies suggest that health impairment is heightened among those traveling abroad (Bauer et al., 2005; Mitchell et al., 2011; Steffen et al., 2003), especially in developing countries (Hill, 2006; Steffen et al., 2003), there has been little discussion within travel medicine and tourism scholarship concerning health care provision in destination locations. Thus, as one of few investigations to specifically examine destination perspectives on providing medical care for ill or injured tourists, this analysis offers a novel addition to a limited body of literature. Further, this study provides insight into the ways in which health care provision is affected by, and intersects with, the tourism sector on Cozumel Island, a destination that continues to be economically dependent upon the daily movement of transnational consumers. Through our analysis of 13 semi-structured interviews with health care providers working on Cozumel, we have identified three key (overlapping) challenges faced when providing care for ill or injured tourists: resource deficiencies, medical (mis)perceptions, and remuneration complexities—and have revealed potential strategies employed in the mitigation of these challenges. Resource deficiencies are understood as challenges for recruiting and retaining specific human resources, as well as obtaining or accessing medical equipment and consumables; medical (mis)perceptions concerns the challenge of legitimizing Cozumel’s care provision in light of contradictory views held by tourists who become patients; and, remuneration complexities are defined as challenges experienced in obtaining payment for care provision.

There are parallels between the challenges of caring for tourists in Cozumel identified in this analysis and findings in existing research. For example, existing studies show that hospitals and clinics located outside of major urban centres typically face equipment shortages (Weinhold & Gurtner, 2014) and that health worker shortages are also common due to providers’ reluctance to work in isolating environments or those that lack social and cultural facilities (Kulig, Kilpatrick, Moffitt, & Zimmer, 2015; Mbemba, Gagnon, & Hamelin-Brabant, 2016). This includes isolated and peripheral places in Mexico (Pelcastre-Villafuerte et al., 2016). Similarly, the cyclical nature of tourism seasonality affects a variety of destination market sectors, complicating access to resources, as well as recruitment and retention of human resources (Terry, 2015; Turrión-Prats & Duro, 2016). Our findings mirror such challenges of equipment and resource access, as well as explanations for recruitment and retention of health care professionals on Cozumel Island. Cozumel Island’s resource challenges also reflect known inequities in Mexico’s health care distribution (Laurell, 2007), reinforcing the characterization of private health care in the country as dominated by “small, badly equipped, and poorly staffed hospitals” (Laurell, 2007, p. 519). Further, challenges in accessing blood products on the island reported by participants echo concern found across local news media (Holguin-Resch, 2017; Wilkinson, 2017) and online tourist communities (TripAdvisor, 2013; Cozumel Hotels, 2008) about the impacts this has on patient health. Existing research also acknowledges the potential for cultural tensions to emerge when accessing health care abroad (Hudson et al., 2016; Whittaker & Chee, 2015), and notes that both Mexico and Mexican people suffer from negative stereotypes of apathy, crime, corruption and underdevelopment (Correa-Cabrera & Garrett, 2014; Lasso & Esquivel, 2014) that have been used to define the Mexican medical system as substandard and dangerous (Dalstrom, 2012). As such, it is not unexpected to find that Cozumel’s tourists corroborate such characterizations of health care in Mexico, believing the island to be a place where medical practice is imbricated with incompetence and underdevelopment, thereby complicating patient decision-making and challenging care providers. Medical tourism destinations can also suffer from similar challenges of perception (Han & Hyun, 2015; Khan, Chelliah, & Haron, 2016), with literature revealing an emphasis on empathetic care practices and facility aesthetics in order to underscore quality and competence in light of potentially harmful perceptions (Cook, 2010; Liu & Chen, 2013; Solomon, 2011). Our study finds similar care and aesthetic decisions being made within Cozumel Island’s health care facilities as a way to mitigate some of the challenges of treating tourist-patients, suggesting a certain transnational uniformity in the priorities of providing medical care for privately paying foreign patients.

Participants noted that in an attempt to alleviate resource deficiency challenges faced by specific hospitals or clinics, there is some degree of resource sharing. While some existing research has emphasized the benefits of health care resource pooling in low-resource settings (Karsten, Slikker, & van Houtum, 2015; Pasin, Jobin, & Cordeau, 2002), applied examples of such sharing remain sparse and generally focus on macro-scale, cross-border care arrangements between public health systems (Galan, Olsavszky, & Vlădescu, 2013; Glinos & Baeten, 2014). These cross-border care examples lack consideration of the competitive nature of private health care. As representatives of competing facilities on Cozumel Island, the resource sharing relationships noted by participants are reasonably unexpected considering the competitive nature of private health care, especially amongst clustered hospitals and clinics that are all vying for international patients (Snyder, Crooks, Johnston, Cerón, & Labonte, 2016). Willingness to lend expertise and equipment to competing hospitals suggests that, for Cozumel Island, such networks of interdependence are of considerable importance for ensuring the practical delivery of appropriate and necessary medical care for tourists. Further, as has been documented within other tourist-dependent communities in Mexico (Adams, Snyder, Crooks, & Berry, 2017), this cooperative competition between Cozumel Island’s health care providers may also be understood as a collective strategy to not only maintain and regulate quality of care, but also in order to protect the reputation of the island’s health care sector against common (mis)perceptions of Mexican health care as inferior and unsafe (Dalstrom, 2012).

As we have shown, the challenges for providing health care for tourists on Cozumel Island, and their associated mitigation strategies, present both similar and unique features when contrasted with existing literature on health care provision in touristic spaces. However, it is important to contextualize these findings within the interplay between Cozumel Island’s historic and contemporary health care sector and the island’s dominant tourism landscape. With lateral development of contemporary health care services and tourism on the island (Hospital Médica San Miguel (MSM), 2017), we contend that the challenges presented by our participants can be understood as part of a continuing legacy of entanglement between these sectors. While above we have noted that the cyclical nature of tourism in some destinations can affect resource capability across numerous sectors, for Cozumel Island the cruise industry creates a daily, in addition to seasonal (Pavón, Acros, Soriano, & Farmer, 2016) transience that contributes to fluctuations in demand for more specialist medical equipment and resources, while affecting health human resources, with hospitals struggling to recruit and retain health workers. For example, participants note that despite the potential of treating higher paying tourists, the cycles of Cozumel Island’s tourism sector can dissuade health workers, especially nurses, from practicing on the island due to lack of social facilities when tourism is “low”. While many tourism destinations may experience such annual fluctuations, the cruise industry’s dominance of Cozumel Island’s tourism sector means that even during the island’s yearly “high season” such cyclical fluctuations of activity and tourist numbers continue on a more micro, daily level. As such, the island experiences a fleeting excitement of tourism by day that, at night, recedes with the cruise ships—leaving few options for health care providers to engage socially outside of employment hours as many entertainment and dining options tend to close with the departing ships. The challenges provided by tourism for the provision of health care are further evident for remuneration of services. With tourists making up the majority of patients within Cozumel Island’s international care facilities, participants indicate that remuneration for services is intricately entangled with complexities of tourism, including access to payments (e.g., complications of communication with insurance companies; inability to pay out-of-pocket), tourist’s poor understanding of insurance coverage, or the navigation of complicated policies. Here, remuneration for treatment relies directly upon travel insurance services or tourists’ preparedness for unforeseen health events, thus imbricating tourism within a challenge which must be overcome by the island’s health care services.

As with the provision of health care, the mitigation of challenges for such provision also exists within the entanglement between Cozumel Island’s health care sector and the island’s dominant tourism landscape. Here we contend that as health care provision can never be fully extricated from the tourism landscape of Cozumel Island, tensions are created for providers that affect care as they must conceptualize both their patients and themselves across the expectations and goals of each sector. Participant rhetoric and action suggests that health care workers are cognizant that patients remain as tourists, and, as they provide treatment services, understand that their provision of care is simultaneously the provision of a tourism service. Thus, care provision itself, while providing necessary medical assistance for patients, remains representative of Cozumel Island’s reputation as a tourism destination, with the mitigation of challenges akin to strategies for customer satisfaction seen elsewhere within tourism services. Practices such as resource sharing between competing hospitals, focusing upon personalized and empathetic patient-centred forms of care, providing culturally familiar communications that assist in the legitimation of practice, and offering familiar care aesthetics exist in part to ensure confidence in quality and safety of the island’s health care services, but also to maintain the reputation of Cozumel Island as a safe, progressive and well-resourced tourist destination. However, it must be noted that the profit-driven nature of Cozumel Island’s private health care cannot always align with such reputational protection strategies, which may be disrupted by complications such as the inability to remunerate services that require providers to stop treatment. For example, stabilizing and then discharging patients who are unable to pay is likely to operate counterintuitive to reputational maintenance or protection activities, thereby potentially creating or reinforcing negative perceptions about the island’s quality of care, and its broader reputation as a tourism destination.

We have suggested that providing medical care for tourists on Cozumel Island involves a number of challenges and mitigation strategies that exist as part of an inseparable entanglement between the island’s private health care sector and its tourism landscape. There are implications of this entanglement for Cozumel Island as a tourism destination. Within ideas shared by the island’s health care professionals there exists a cognizance of the patient’s origin as tourist and a belief that although now requiring medical attention, patients remain as tourists. Further, their rhetoric and actions suggest an understanding that the island’s private health care sector operates as an ancillary tourism service, remaining necessary to sustain Cozumel Island’s economic livelihood as a tourism destination (and vice-versa). While an unforeseen health event will contribute to a tourist’s admission to one of the island’s private hospitals, the results of this study propose that the island’s health care providers understand that a tourist’s health care experience continues to be part of *what happened on holiday*. Thus, it is here that we suggest an imperative for tourism providers, such as cruise lines who each day unload the majority of Cozumel Island’s soon-to-be foreign patients, to consider their accountability to this ancillary tourism sector contributing to the continued success of Cozumel Island as a popular port of call in the Western Caribbean and overnight holiday destination. It is in the best interests of such providers to ensure that their customers are both aware of the level of care provided in Cozumel’s hospitals, and are equipped with the necessary means to remunerate any services procured upon the island.

Within this article we have identified a number of challenges faced in providing health care for tourists on Mexico’s Cozumel Island, as revealed by health workers in the island’s private health care sector. While challenges are interwoven throughout issues of resource availability, medical (mis)perceptions, and remuneration complexities, of particular note is that such challenges exist within an entanglement between Cozumel Island’s health care sector and the island’s tourism landscape. With little literature to provide comparison, it remains difficult to ascertain if the entanglements and challenges within this study may accurately describe other tourism destinations. Similar research in other tourism-dependent areas is thus useful. Further, Cozumel Island offers unique geographies of (in)accessibility not found in similarly energetic tourism areas given its distance from mainland Mexico. Thus, a number of future research avenues are worth mentioning. To assess transferability of the findings reported here, it is important to investigate health care in other similarly tourism-dependent destinations, especially those dominated by the cruise industry. Research should continue to uncover what it means to practice within a health care sector reliant on tourism, and, critically, include focus on the patient experience to provide an alternative perspective of engagement with health care on holiday. Finally, with manifold literature reminding us of tourism’s penchant for unfavourable outcomes, we broadly call for researchers to pay significantly more attention to the nexus of health care and tourism within destination locations.

## Conclusion

This article has examined the challenges faced, and mitigation strategies employed, by health care workers on Cozumel Island, Mexico, as they provide medical treatment to ill and injured tourists. Analyzing the findings of semi-structured interviews conducted with 15 health care providers on Cozumel, we have identified three key challenges for care provision: resource deficiencies, the (mis)perception of the island’s health care sector, and complexities for remuneration. We have also shown that participants employ specific strategies in the mitigation of these challenges, including the sharing of both human and material resources, as well as a focus on providing empathetic care and culturally familiar interactions and environments for tourists. Complicating our findings, we suggest that both the challenges faced, and the mitigation practices employed, exist within an entanglement between Cozumel Island’s health care sector and the island’s broader touristic landscape. Within this entanglement, tensions for conceptualizing both patients and providers exist, leading to health care provision that simultaneously seeks to provide medical attention for tourists while maintaining the reputation of Cozumel Island as a tourism destination. Understanding the importance of Cozumel Island’s health care sector to its touristic aspirations, we believe it is important for tangential tourism providers, such as the cruise lines which service the island, to take a larger role in the mitigation of challenges for health care provision on Cozumel Island.
